# A flavanone from *Baccharis retusa* (Asteraceae) prevents elastase-induced emphysema in mice by regulating NF-κB, oxidative stress and metalloproteinases

**DOI:** 10.1186/s12931-015-0233-3

**Published:** 2015-06-30

**Authors:** Laura Taguchi, Nathalia M. Pinheiro, Clarice R. Olivo, Alessandra Choqueta-Toledo, Simone S. Grecco, Fernanda D.T.Q.S. Lopes, Luciana C. Caperuto, Mílton A. Martins, Iolanda F.L.C. Tiberio, Niels O. Câmara, João Henrique G. Lago, Carla M. Prado

**Affiliations:** Department of Biological Science, Universidade Federal de São Paulo, Rua Artur Riedel, 275 - Eldorado, Diadema, SP Brazil; Department of Exact and Earth Sciences, Universidade Federal de São Paulo, Diadema, Brazil; Department of Medicine, Faculdade de Medicina da Universidade de São Paulo, São Paulo, Brazil; Department of Immunology, Biological Institute, Universidade de São Paulo, São Paulo, Brazil

**Keywords:** Emphysema, Flavonoid, NF-κB, Oxidative stress, Lung remodeling, Experimental models

## Abstract

**Background:**

Pulmonary emphysema is characterized by irreversible airflow obstruction, inflammation, oxidative stress imbalance and lung remodeling, resulting in reduced lung function and a lower quality of life. Flavonoids are plant compounds with potential anti-inflammatory and antioxidant effects that have been used in folk medicine. Our aim was to determine whether treatment with sakuranetin, a flavonoid extracted from the aerial parts of *Baccharis retusa,* interferes with the development of lung emphysema.

**Methods:**

Intranasal saline or elastase was administered to mice; the animals were then treated with sakuranetin or vehicle 2 h later and again on days 7, 14 and 28. We evaluated lung function and the inflammatory profile in bronchoalveolar lavage fluid (BALF). The lungs were removed to evaluate alveolar enlargement, extracellular matrix fibers and the expression of MMP-9, MMP-12, TIMP-1, 8-iso-PGF-2α and p65-NF-κB in the fixed tissues as well as to evaluate cytokine levels and p65-NF-κB protein expression.

**Results:**

In the elastase-treated animals, sakuranetin treatment reduced the alveolar enlargement, collagen and elastic fiber deposition and the number of MMP-9- and MMP-12-positive cells but increased TIMP-1 expression. In addition, sakuranetin treatment decreased the inflammation and the levels of TNF-α, IL-1β and M-CSF in the BALF as well as the levels of NF-κB and 8-iso-PGF-2α in the lungs of the elastase-treated animals. However, this treatment did not affect the changes in lung function.

**Conclusion:**

These data emphasize the importance of oxidative stress and metalloproteinase imbalance in the development of emphysema and suggest that sakuranetin is a potent candidate that should be further investigated as an emphysema treatment. This compound may be useful for counteracting lung remodeling and oxidative stress and thus attenuating the development of emphysema.

**Electronic supplementary material:**

The online version of this article (doi:10.1186/s12931-015-0233-3) contains supplementary material, which is available to authorized users.

## Background

The main feature of chronic obstructive pulmonary disease (COPD) is pulmonary airflow restriction that is not fully reversible; this limitation is usually progressive and is associated with an abnormal inflammatory response to the inhalation of noxious particles and gases. COPD is usually initiated by exposure to aggressive agents, such as cigarette smoke, which induce secretory gland hypertrophy and hyperplasia as well as mucus hypersecretion; these are characteristics of chronic bronchitis, a component of COPD [1]. In the periphery, chronic airway inflammation induces a repetitive process of injury and repair, leading to structural alterations in airways. Three states of COPD have been well described; these states can occur concomitantly or separately and include narrowed airway thickness, limited expiratory airflow, and increased production of secretions, which induces chronic cough and emphysema. Indeed, it is difficult to differentiate between chronic bronchitis and emphysema because they often coexist in COPD [1].

Emphysema, which develops in 15 % to 20 % of smokers and 1 % of non-smokers [2], is defined as an airspace enlargement that is usually induced by oxidative stress and an imbalance between proteases and antiproteases that leads to inflammatory processes. In turn, a sequential cascade of responses is triggered that culminate in tissue destruction [3]. Several proinflammatory cytokines and chemokines, such as tumor necrosis factor (TNF)-α, interleukin (IL)-1β, macrophage inflammatory protein (MIP)-2 and monocyte chemoattractant protein (MCP)-1, as well as certain growth factors such as macrophage colony-stimulating factor (M-CSF), are involved in COPD physiopathology and contribute to maintaining the inflammation [2]. The transcription factor nuclear factor-kappa B (NF-κB) controls the expression of many of the inflammatory and apoptotic genes present in COPD [4].

Macrophages and neutrophils release matrix metalloproteases (MMPs), which are proteolytic enzymes, and tissue inhibitors of metalloproteinases (TIMPs). In emphysema, the expression of MMP-9 and MMP-12 is increased [5]. Destruction and repair of the lung parenchyma occur together in emphysema, and the observed destruction could be, at least in part, a consequence of misguided repair. The turnover of extracellular matrix fibers is continuous and results from the migration and persistence of inflammatory cells recruited by the protease/antiprotease imbalance and oxidative stress.

Reactive oxygen species (ROS) and nitrogen species are involved in the development of COPD. It is well known that, in COPD, there is not only an increase in oxidants production but also an important reduction in the anti-oxidative potential since the levels of some antioxidant can be significantly diminished [6, 7]. Antioxidants ingested through the diet and supplements can remove ROS and provide health benefits [8]. Many studies have shown that vegetables are rich sources of antioxidant compounds, such as phenols, flavonoids, quinones, and vitamins, that may decrease the incidence of diseases associated with oxidative stress [9, 10].

Flavonoids, which are low molecular weight aromatic compounds with up to 15 carbon atoms in the basic skeleton, are natural pigments found in many plants [8, 11] and are widely consumed. Some of these compounds can be found in specimens of the *Baccharis* genus, which is represented by more than 500 species distributed primarily in the tropical areas of South America, including Brazil. Many of these species are extensively used in folk medicine for the treatment or prevention of anemia, diabetes, stomach and liver diseases, and inflammation [12]. Several different flavonoids have been described in these species [13], including sakuranetin (5,4’-dihydroxy-7-methoxyflavanone), which was previously reported to be the main component of the aerial parts of *B. retusa* [14]. Our group recently showed that sakuranetin has potential *in vitro* antileishmanial and antitrypanosomal activity [15] as well as anti-inflammatory activity in an *in vivo* experimental asthma model [16].

Currently, the recommended treatment for COPD involves controlling the symptoms, reducing exacerbations and improving the quality of life, but these interventions do not reverse lung emphysema or even prevent disease progression [17]. Sakuranetin is a potential antioxidant because it is a flavanone with reported anti-inflammatory activity. We therefore hypothesized that sakuranetin would interfere with emphysema physiopathology.

To investigate the anti-inflammatory and antioxidant effects of flavonoids in emphysema, we used an experimental model of emphysema. In this model, emphysema is induced *via* the instillation of elastase into C57BL6 mice. The present study showed reductions in lung inflammation, which was associated with attenuated lung parenchymal remodeling, and in alveolar destruction in the sakuranetin-treated emphysematous animals. These effects appeared to be mediated through the inhibition of NF-κB. Our findings highlight the importance of counteracting oxidative stress and metalloproteinase activity in the physiopathology of emphysema and provide insights into a new therapeutic modality for treating emphysema, lung inflammation and oxidative stress in cases that are not controlled by conventional treatments.

## Methods

### Ethics statement

This study was approved by the Ethics Committees of UNIFESP (CEP; protocol number 0982/10) and USP (CAPPESQ; protocol number 307/10). All the animals received humane care according to the "Guide for the Care and Use of Laboratory Animals" (Institute of Laboratory Animal Resources - 1996), and all the surgical procedures were performed while the animals were under general anesthesia. The mice used in this study were maintained in a temperature-controlled room at 21–23 °C with a 12-hour light/dark cycle and *ad libitum* access to water and food.

### *Baccharis retusa* collection and extraction/isolation of sakuranetin

The aerial portions of *Baccharis retusa* DC. were collected in Campos do Jordão/SP, Brazil, in October 2011. The plant material was identified by Dr. Oriana A. Fávero and compared with a voucher specimen (SPSF44897) deposited at the Herbario D. Bento Pickel, Instituto Florestal de São Paulo (IF/SP), São Paulo, Brazil.

Powdered, air-dried aerial parts of *B. retusa* (500 g) were defatted with hexane (4 X 500 mL) and exhaustively extracted with MeOH at room temperature. After partial removal of the solvent, the extract was resuspended in MeOH:H_2_O (1:2 v/v) and extracted successively with hexane, CH_2_Cl_2,_ and EtOAc. The CH_2_Cl_2_ phase was subjected to column chromatography over silica gel (230–400 mesh; Merck, Kenilworth, NJ, USA) and eluted with CH_2_Cl_2_ containing increasing amounts of EtOAc (up to 100 %) to produce six fractions (A1–A6). Fraction A2 (712 mg) contained pure sakuranetin, which was analyzed by nuclear magnetic resonance (NMR; ^1^H and ^13^C) and low-resolution electronic impact mass spectrometry (LREIMS) (see Additional file 1).

### Experimental groups

A total of 30 C57BL6 male mice aged 7–9 weeks and weighing 25 g were randomly assigned to one of 4 groups: a, saline and sakuranetin (SAL+SK group, *n* = 7); b, elastase and sakuranetin (ELA+SK group, *n* = 8); c, saline and DMSO vehicle (SAL+Ve group, *n* = 7); and d, elastase and DMSO vehicle (ELA+Ve group, *n* = 8). Another set of 30 animals were assigned to the groups described above (values of n were the same as described above) and were treated similarly; these animals were used only for evaluating lung mechanics, after which the lungs were removed and frozen. At the end of the protocol, the animals were 11 to 13 weeks old. DMSO was used to dilute sakuranetin because it is insoluble in pure saline.

### Protocol for elastase-induced emphysema

The animals were anesthetized with an intramuscular injection of ketamine (40 mg/kg) and xylazine (5 mg/kg) before the infusion of porcine pancreatic elastase (PPE) (day zero). The ELA+SK and ELA+Ve groups received 50 μL of elastase (6.6 units/mg, E1250, Type 1; Sigma, St. Louis, MO) *via* an intranasal drop (0.677 IU) [18]. The animals in the SAL+SK and SAL+Ve groups received an intranasal instillation of the same volume of saline.

### Protocol for sakuranetin treatment

The animals in the SAL+SK and ELA+SK groups were treated with sakuranetin by intranasal instillation on day 0 (2 h after the instillation of saline or elastase) and on days 7, 14 and 28 of the experimental protocol. The dose of 20 mg/kg diluted in 10 μL of DMSO:saline (1:4) was selected based on previous studies that used this compound in other experimental models [16]. The control animals (SAL+Ve and ELA+VE groups) were treated with vehicle *via* the same method.

### Respiratory mechanics evaluation

Thirty minutes after the last sakuranetin treatment and 28 days after the elastase instillation, the animals were deeply anesthetized with an intraperitoneal injection of thiopental (70 mg/kg), tracheotomized and then connected to a small animal ventilator (FlexiVent, SCIREQ, Montreal, Canada). The animals were ventilated at 150 breaths/min with a tidal volume of 10 mL/kg, and a positive end expiratory pressure (PEEP) of 5 cm H_2_O was applied. Vcyl was corrected to obtain the actual volume in the animals (V), and Pcyl was corrected based on the value of Pao (open airway pressure). We obtained the flow (V') by the derivation of V with respect to time. We analyzed the respiratory system resistance (Rrs) and elastance (Ers) as previously described [16]. The experimental data from the forced oscillation technique were obtained only after the animals had been paralyzed with pancuronium bromide (0.2 mg/kg). Oscillatory lung mechanics were analyzed by producing flow oscillations at different frequencies (from 0.25 to 19.625 Hz) for 16 s. The pressure and flow data were obtained, and the airway impedance was calculated at each frequency. Airway resistance [19], tissue damping (Gtis) and tissue elastance (Htis) parameters were obtained by applying the constant phase model [20].

### Bronchoalveolar lavage fluid (BALF)

At the end of the mechanical evaluation, the anterior chest wall was opened, the animals were exsanguinated *via* the abdominal aorta, and the BALF was collected [16]. The trachea was cannulated, and the BALF was obtained by washing the airway lumen with 0.5 mL of sterile saline and withdrawing the fluid. This procedure was repeated three times. The recovered volume was greater than 95 % of the instilled fluid, and the fluid was transferred to a test tube on ice. For total and differential cell counting, the BALF was centrifuged (Model GS-6R Centrifuge, Beckman Instruments, Fullerton, USA) at 800 x g for 8 min at 5 °C, and the cell pellet was resuspended in 0.2 mL of sterile saline. The total number of viable cells was determined using a *Neubauer* hemocytometer and an optical microscope (Model BX40 Olympus Optical Co. Tokyo, Japan) at 400X magnification. Differential cell counting was performed on cytocentrifuge preparations of BALF (450 rpm for 6 min) (model: Cytospin 3, Shandon Instruments, Sewickley, USA) on glass slides, which were stained with Diff-Quick (Biochemical Sciences Inc., Swedesboro, NJ). At least 300 cells were differentiated according to the standard morphologic criteria at a magnification of 1000X using the optical microscope described above.

### Morphometric studies

After collecting the BALF, the lungs were removed in blocks, fixed using 4 % buffered formalin infused through the trachea at a constant pressure of 20 cm H_2_O for 24 h (to maintain a similar alveolar histoarchitecture among the groups), and embedded in paraffin. Histological sections (3–5 μm thickness) were cut and subjected to morphometric analysis.

### Mean linear intercept (Lm)

Lung tissue sections from five animals per group were stained with hematoxylin and eosin (H&E) to evaluate the mean linear intercept, an index that is widely used to characterize the presence of emphysema [18]. For this analysis, an eyepiece with a coherent system of 50 lines, 100 points, and a known area was attached to the microscope ocular (Model BX40 Olympus Co). The mean linear intercept (Lm) was assessed in 20 randomly selected, non-overlapping fields of the distal lung parenchyma in each animal at a magnification of 200X, and we counted the number of times that the lines of the integrating eyepiece intersected with the alveolar septum.

### Extracellular matrix (ECM) remodeling

Picrosirius (Direct Red 80, CI 35780; Sigma Aldrich, Milwaukee, WI) was used to quantify collagen fibers, and Weigert’s resorcin-fuchsin with oxidation was used to detect elastic fibers. A Leica DM4000B microscope (Leica Microsystems, Wetzlar, Germany), a digital camera (Leica DFC420 Leica Microsystems) and Image-Pro Plus 4.5 image analysis software (Image-Pro Plus for Windows 98/NT/2000 4.5.0.29, Media Cybernetics Inc., Bethesda, MD, USA) were used to evaluate collagen and elastic fiber deposition in the alveolar walls. Ten randomly selected fields from each animal were evaluated at 400X magnification. The collagen and elastic fiber area was expressed as a percentage of the total alveolar wall area [16].

### Immunohistochemistry

Immunohistochemical staining was performed with an antibody against the isoprostane 8-iso-PGF2α (1:10,000 dilution; Oxford Biomedical Research, Rochester Hills, MI) [16] as an indirect method to evaluate oxidative stress. The sections were deparaffinized and washed seven times for 5 min with 3 % H_2_O_2_ to inhibit endogenous peroxidase activity. After the PBS and water washes, antigen retrieval was performed with trypsin for 20 min. Then, the sections were washed in PBS three times for 3 min each and incubated overnight with the anti-8-iso-PGF2α antibody diluted in BSA. After the sections were washed with PBS, the reagent from the Vectastain ABC Kit (Vector Elite PK-6105, Burlingame, USA) was used as the secondary antibody, and DAB (Sigma-Aldrich) was used as the chromogen. The sections were counterstained with Harris hematoxylin (Merck, Darmstadt, Germany). The image analysis of the 8-iso-PGF-2α-stained slides was performed as described previously [16]. The 8-iso-PGF-2α-positive area in the lung was expressed as a percentage of the total area of the lung tissue.

### Detection of cells positive for MMP-9, MMP-12, TIMP-1 and p65-NF-κB

Immunohistochemical staining was performed using goat polyclonal IgG anti-MMP-9 (SC6840, 1:500 dilution), goat polyclonal IgG anti-MMP-12 (SC8839, 1:1.200 dilution), rabbit polyclonal IgG anti-TIMP-1 (SC 5538, 1:100 dilution), and rabbit polyclonal IgG anti-p65-NF-κB (SC109, 1:300 dilution) antibodies (Santa Cruz Biotechnology, Santa Cruz, CA) using the same biotin–streptavidin–peroxidase method described above. We determined the number of cells positive for MMP-9, MMP-12, TIMP-1 and p65-NF-κB using the point-counting technique as described above. Twenty randomly selected fields of the alveolar wall were analyzed in each animal at 1000X magnification. The results are expressed as the number of positive cells per unit area (10^4^ μm^2^) [16].

### Bio-Plex

The lungs were removed and quickly frozen to obtain cytokine measurements in the lung homogenate. A Bio-Plex Mouse Cytokine Assay Kit (Bio-Rad Laboratories, Inc., Hercules, CA, USA) was used to determine the presence of 5 cytokines in the lung homogenates. The assay was performed using the Bio-Plex suspension array system, and the data were analyzed using Bio-Plex Manager software version 4.0. The standard curves ranged from 32,000 to 1.95 pg/mL, as previously described [16]. The values for M-CSF, TNF-α, IL-1β, MCP-1 and MIP-2 in the lung homogenates were expressed as pg cytokine/mg total protein.

### p65-NF-κB protein analysis by Western blotting

Lung fragments weighing approximately 100 mg were homogenized in a boiling extraction buffer [10 % SDS, 100 mM Tris (pH 7.4), 10 mM EDTA, 10 mM sodium pyrophosphate, 100 mM sodium fluoride, and 10 mM sodium vanadate] with a Polytron PTA 20S generator (model PT 10/35, Brinkmann Instruments, Inc., Westbury, NY) at maximum speed for 30 s. The extracts were centrifuged at 15,000 x g for 15 min at 4 °C to remove the insoluble material. The protein concentrations in the supernatants were determined using the Bradford assay, and an equal amount of total protein from each sample (75 μg) was treated with Laemmli buffer containing 100 mM dithiothreitol. The samples were heated in a boiling water bath for 5 min and then subjected to SDS-PAGE (10 % bis-acrylamide).

The proteins were electrotransferred from the gel to a nitrocellulose membrane over 90 min at 120 V (constant) as described previously [21]. Nonspecific protein binding to nitrocellulose was reduced by preincubating the membrane overnight at 4 °C in blocking buffer (2.5 % milk/TBST). The nitrocellulose membrane was then incubated overnight at 4 °C with anti-p65-NF-κB (1:400; Santa Cruz Biotechnology, Santa Cruz, CA) and anti-β-actin (1:1000; Sigma Aldrich, St. Louis, MO) antibodies diluted in blocking buffer and washed for 30 min with TBST.

The bound antibodies were detected with horseradish peroxidase-conjugated (HRP-conjugated) anti-IgG (1:10,000) and visualized using chemiluminescence with UVItec (UVItec Limited, Cambridge, UK). The band intensities were quantified using the UVItec Image Program.

### Statistical analysis

The data were analyzed using Sigma Stat 10 (SPSS Inc., CA, USA). Normality was evaluated using the Kolmogorov-Smirnov test, and the data are presented as the mean ± SE. The parametric data were analyzed by two-way ANOVA (with elastase treatment and sakuranetin treatment as the two parameters) followed by the Holm-Sidak test. Differences with *p* < 0.05 were considered significant.

## Results

### Chemical characterization of sakuranetin

The isolated flavonoid was characterized based on analysis by NMR (^1^H and ^13^C) and LREIMS (see Additional file 1). The spectroscopic data were compared with those reported in the literature [16] to confirm the identification of sakuranetin (5,4′-dihydroxy-7-methoxyflavanone).

### Vehicle treatment (DMSO plus saline) did not affect lung responses

Because DMSO is an organo-sulfur compound that possesses both toxic and antioxidant effects, we first evaluated whether DMSO treatment (vehicle) interfered with lung inflammation and 8-iso-PGF-2α expression by comparing vehicle-treated animals (DMSO+Saline) with untreated animals. There were no significant differences in lung inflammation or in the 8-iso-PGF-2α-positive area (evaluated by *t*-test) between animals treated with or without vehicle (see Additional file 1). Therefore, we used the vehicle-treated animals as controls for sakuranetin treatment. We previously evaluated the effects of DMSO in other models of lung disease and did not observe any effects [16].

### Sakuranetin treatment did not affect lung elastance

**Fig. 1:**
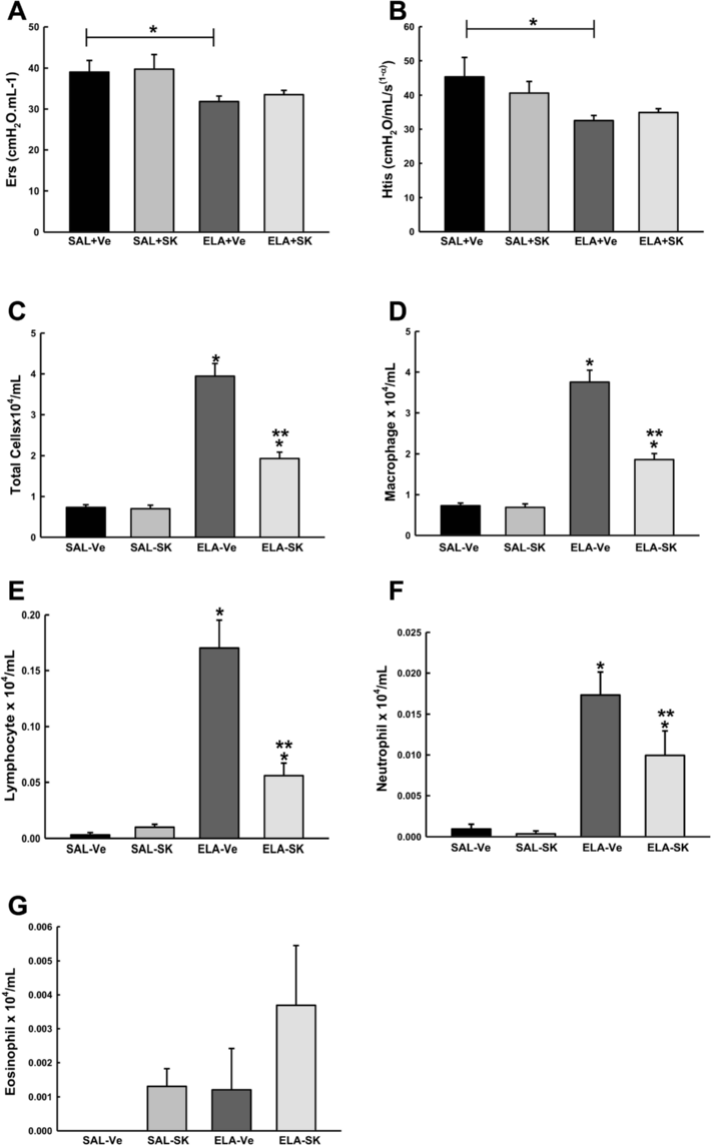
Lung mechanics and lung inflammation. The mean and standard error (SE) of respiratory system elastance (Ers) **a** and lung tissue elastance (Htis) **b** evaluated using a Flexivent ventilator under anesthesia 28 days after elastase or saline instillation. After the lung mechanics were evaluated, the animals were exsanguinated, and bronchoalveolar lavage fluid was collected. We quantified total cells **c**, macrophages **d**, lymphocytes **e**, neutrophils **f** and eosinophils **g** in the four groups. SAL+Ve: control group with vehicle treatment; ELA+Ve: elastase instillation and vehicle treatment; SAL + SK: control group treated with sakuranetin; ELA+SK: elastase- and sakuranetin-treated group. *p < 0.05 compared with the control (SAL+Ve and SAL+SK); **p < 0.05 compared with the ELA+Ve group

The Ers and Htis values are presented in Fig. 1 (a and b, respectively). The ELA+Ve group exhibited lower Ers values compared with the SAL+Ve group (*p* < 0.05). Sakuranetin treatment had no effect on Ers compared with the vehicle-treated elastase-instilled animals since ELA+SK showed; similar results than SAL+Ve and ELA+Ve groups. We observed a reduction in Htis (Fig. 1b) in the ELA+Ve group compared with the control groups (SAL+Ve and SAL+SK) (*p* < 0.05). Sakuranetin treatment had no effect on Htis in the elastase-treated groups.

There were no significant differences in the Rrs, Gtis and Raw values among the experimental groups (data not shown).

### Sakuranetin treatment reduced lung inflammation and pro-inflammatory cytokine levels in lung homogenates

In Fig. 1c-g, we present the number of total cells (c), macrophages (d), lymphocytes (e), neutrophils (f) and eosinophils (g). The animals that received elastase and were treated with vehicle (ELA+Ve) or sakuranetin (ELA+SK) showed an increase in total cell number and in the number of macrophages, neutrophils and lymphocytes compared with the control groups (*p* < 0.001 for all comparisons). The number of total cells, macrophages, lymphocytes and neutrophils was reduced in the elastase-instilled animals treated with sakuranetin (ELA+SK) compared with the elastase-instilled, vehicle-treated animals (*p* < 0.05). There were no differences in the number of eosinophils among the experimental groups (Fig. 1g).

The cytokine concentrations in the lung homogenates (pg of cytokine/mg of total protein) were measured using the Bio-Plex system (Table 1). The ELA group had higher levels of M-CSF, IL-1β and TNF-α compared with the control groups (*p* < 0.05). In addition, sakuranetin treatment reduced the levels of these cytokines (*p* < 0.01). Although there was a tendency (*p* = 0.08) for increased MCP-1 expression in the ELA+Ve group, there were no significant differences in MIP-2 and MCP-1 levels among the experimental groups.

**Table 1:** Effects of sakuranetin treatment on cytokine release in lung homogenates as quantified by ELISA (Bio-Plex)

	SAL+Ve	ELA+Ve	SAL+SK	ELA+SK
M-CSF (pg/mL)	1.08 ± 1.05	10.95 ± 1.36*	1.64 ± 1.37	1.72 ± 1.18**
IL-1β (pg/mL)	0.92 ± 0.18	1.92 ± 0.25*	1.28 ± 0.25	0.82 ± 0.22**
MCP-1 (pg/mL)	5.90 ± 2.32	11.00 ± 2.32	4.73 ± 2.68	8.77 ± 2.32
TNFα (pg/mL)	0.21 ± 0.03	0.33 ± 0.04*	0.15 ± 0.04	0.15 ± 0.03**
MIP-2 (pg/mL)	10.00 ± 1.50	10.24 ± 1.50	7.98 ± 1.93	10.55 ± 1.50

Data are presented as the mean ± SE. SAL+Ve: control group with vehicle treatment; ELA+Ve: elastase instillation and vehicle treatment; SAL+SK: control group treated with sakuranetin; ELA + SK: elastase- and sakuranetin-treated group. *p < 0.05 compared with the SAL groups; **p < 0.01 compared with the ELA+Ve group

### Sakuranetin treatment reduced the elastase-induced emphysema

**Fig. 2:**
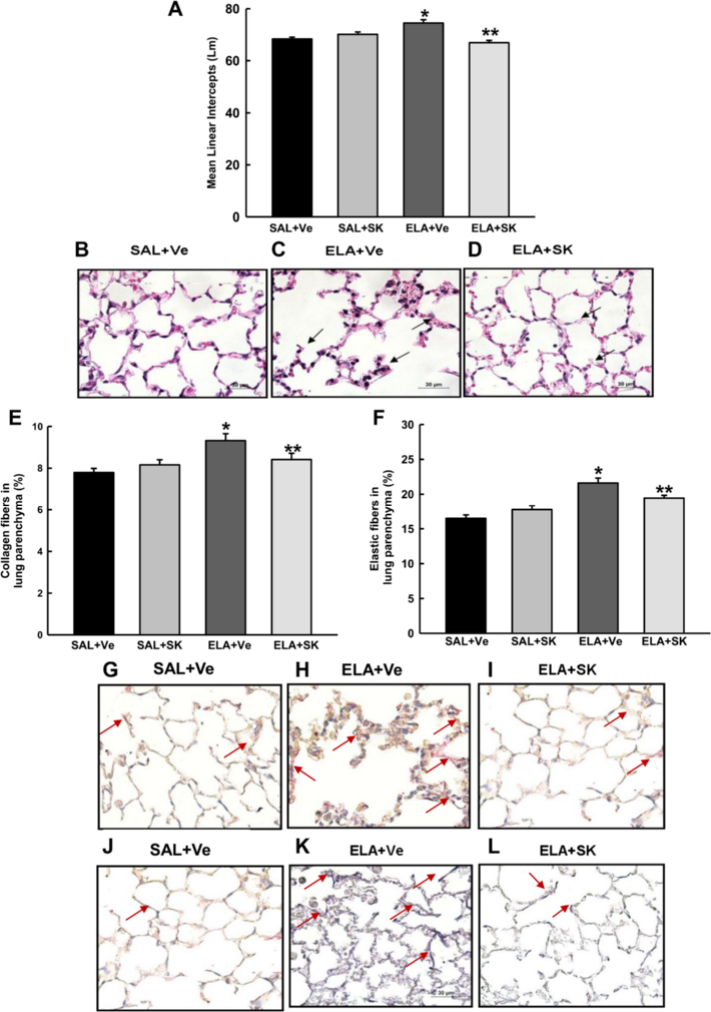
Mean alveolar diameter (Lm) and pulmonary remodeling. The data are presented as the mean and SE for the four groups. The Lm **a**, which correlates with alveolar destruction, was increased in the ELA+Ve group **c** compared with the control group **b**. Sakuranetin treatment attenuated this response in ELA+SK animals **d**. Pulmonary remodeling was characterized by collagen **e** and elastic **f** fiber content in the alveolar septum. There was increased deposition of collagen and elastic fibers in the alveolar septum in the animals that received elastase (collagen **h** and elastic **k** fibers). Sakuranetin treatment (ELA+SK) attenuated this response (collagen **i** and elastic **l** fibers) and generated values that were similar to those in the control group **g** represent collagen and **j** elastic fibers). Collagen and elastic fibers are indicated by arrows. *p < 0.001 compared with the SAL+Ve group; **p < 0.001 (A) and **p < 0.05 (E) compared with the ELA+Ve group

Lm was evaluated as an indicator of lung tissue destruction. The Lm values in the four experimental groups are presented in Fig. 2a. Lm increased in the animals that received elastase and vehicle (ELA+Ve) compared with the animals that received saline and vehicle (SAL+Ve) (*p* < 0.001). Sakuranetin treatment attenuated the emphysema, as indicated by the lower Lm in the ELA+SK group compared with the elastase-instilled, vehicle-treated group (*p* < 0.001). Sakuranetin treatment had no effect in the control groups. Photomicrographs of lung tissue from representative animals from the SAL+Ve (b), ELA+Ve (c) and ELA+SK (d) groups are presented in Fig. 2 (Fig. 2b-d). We observed intense alveolar destruction associated with the presence of inflammatory cells in the ELA+Ve group (arrows, Fig. 2c). These responses were diminished in the elastase-instilled, sakuranetin-treated animals (arrows, Fig. 2c).

### Sakuranetin treatment reduced alveolar remodeling by interfering with the metalloprotease and TIMP balance

The quantification of collagen (e) and elastic (f) fibers in the lung parenchyma is shown in Fig. 2e-l. There were more collagen and elastic fibers in the lung parenchyma from the elastase-instilled, vehicle-treated animals (ELA+Ve) compared with the lung parenchyma from the control animals (SAL+Ve and SAL+SK) (*p* < 0.001 for all comparisons). Sakuranetin treatment reduced both collagen (*p* < 0.05) and elastic (*p* < 0.01) fiber deposition in elastase-treated animals (ELA+SK vs ELA+Ve; *p* < 0.05).

Figure 2g-l presents representative photomicrographs of lung tissue from animals in the SAL+Ve (g and j), ELA+Ve (h and k) and ELA+SK (i and l) groups that was stained for collagen (g-i) or elastic (j-l) fibers. We observed increased collagen and elastic fiber deposition in the ELA+Ve animals (arrows, Fig. 2h and k). This response was attenuated by sakuranetin treatment (arrows, Fig. 2i and l).

**Fig. 3:**
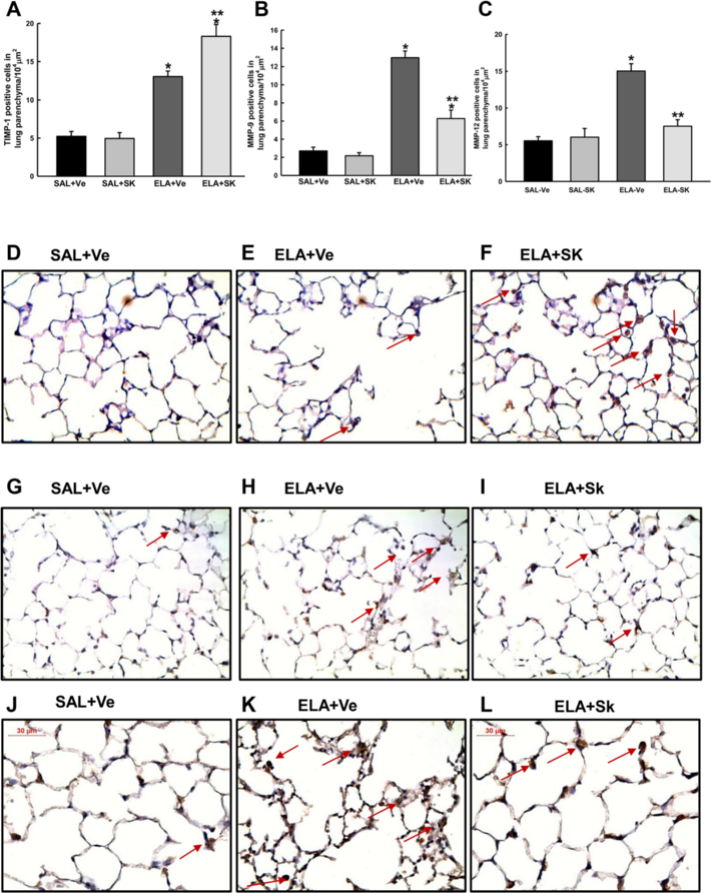
Expression of MMP-9, MMP-12 and TIMP-1 in lung tissue. The mean and SE of the number of cells in the lung parenchyma that were positive for TIMP-1 **a**, MMP-9 **b** and MMP-12 **c**. Representative photomicrographs of lung parenchyma immunostained for TIMP-1 **d-f**, MMP-9 **g-i** and MMP-12 **j-l**. The number of positive cells for TIMP-1, MMP-9 and MMP-12 increased in animals that received elastase **e**, **h** and **k** compared with control animals **d**, **g** and **j**. Interestingly, sakuranetin increased the number of TIMP-1-positive cells and reduced the number of MMP-9- and MMP-12-positive cells in the elastase-treated groups **f**, i and **l**; see arrows). *p < 0.001 compared with the SAL+Ve and SAL+SK groups; **p < 0.05 compared with the ELA+Ve group

Because the degradation and turnover of collagen and elastic fibers are controlled by the balance between MMPs and TIMPs, we used immunohistochemistry to analyze the expression of TIMP-1, MMP-9 and MMP-12 (Fig. 3). We observed an increase in the number of cells that were positive for MMP-9, MMP-12 and TIMP-1 in the ELA+Ve group compared with the control groups (SAL+Ve and SAL+SK) (*p* < 0.001 for all comparisons). Sakuranetin treatment reduced the number of MMP-9- and MMP-12-positive cells in the elastase-treated animals compared with the vehicle-treated animals (*p* < 0.001), although the number of MMP-9-positive cells was also higher compared with control (*p* < 0.001). In addition, sakuranetin treatment increased the number of TIMP-1-positive cells in the ELA+SK animals compared with the ELA+Ve animals (*p* < 0.001). There were no differences in any of these parameters between the control groups.

Figure 3d-l presents photomicrographs of lung tissue immunostained for TIMP-1 (d-f), MMP-9 (g-i) and MMP-12 (j-l) from the SAL+Ve (d, g and j), ELA+Ve (e, h and k) and ELA+SK (f, i and l) groups.

### Sakuranetin treatment reduced oxidative stress, measured as 8-iso-PGF-2α, in lung tissue

**Fig. 4:**
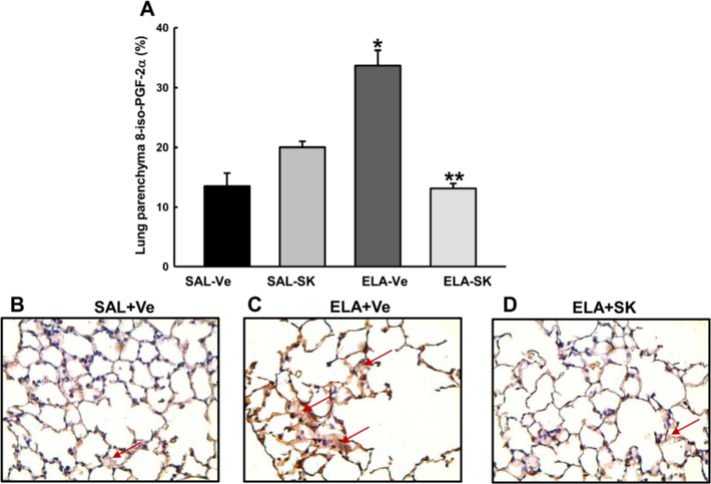
Oxidative stress in lung tissue. 8-iso-PGF-2α-positive area (mean and SE) within the lung parenchyma **a**. Representative photomicrographs of the lung parenchyma immunostained for 8-iso-PGF-2α **b-d**. Vehicle-treated animals that received elastase showed an increase in the 8-iso-PGF-2α-positive area **c** compared with animals in the control group **b**. This response was attenuated in elastase-treated animals that received sakuranetin treatment **d** compared with the ELA+Ve animals **c**. Arrows represent the isoprostane-positive area. *p < 0.001 compared with the SAL+Ve group; **p < 0.001 compared with the ELA+Ve group

In Fig. 4a-d, we observed an increase in the 8-iso-PGF-2α-positive area in vehicle-treated animals that received elastase (ELA+Ve) compared with the saline control animals (*p* < 0.001). Treating the elastase-instilled animals with sakuranetin (ELA+SK) reduced the 8-iso-PGF-2α-positive area compared with the ELA+Ve group (*p* < 0.001). There were no significant differences in oxidative stress between the control groups.

Figure 4d-d presents photomicrographs of 8-iso-PGF-2α-stained lung tissue from the SAL+Ve (b), ELA+Ve (c) and ELA+SK (d) groups. We observed an obvious increase in the 8-iso-PGF-2α-positive area in the alveolar septa of the elastase-instilled animals (c) compared with the saline-instilled animals (b). Sakuranetin treatment markedly reduced the positive staining (arrows, Fig. 4d).

### Sakuranetin treatment reduced the number of NF-κB-positive cells in lung tissue and NF-κB protein expression

**Fig. 5:**
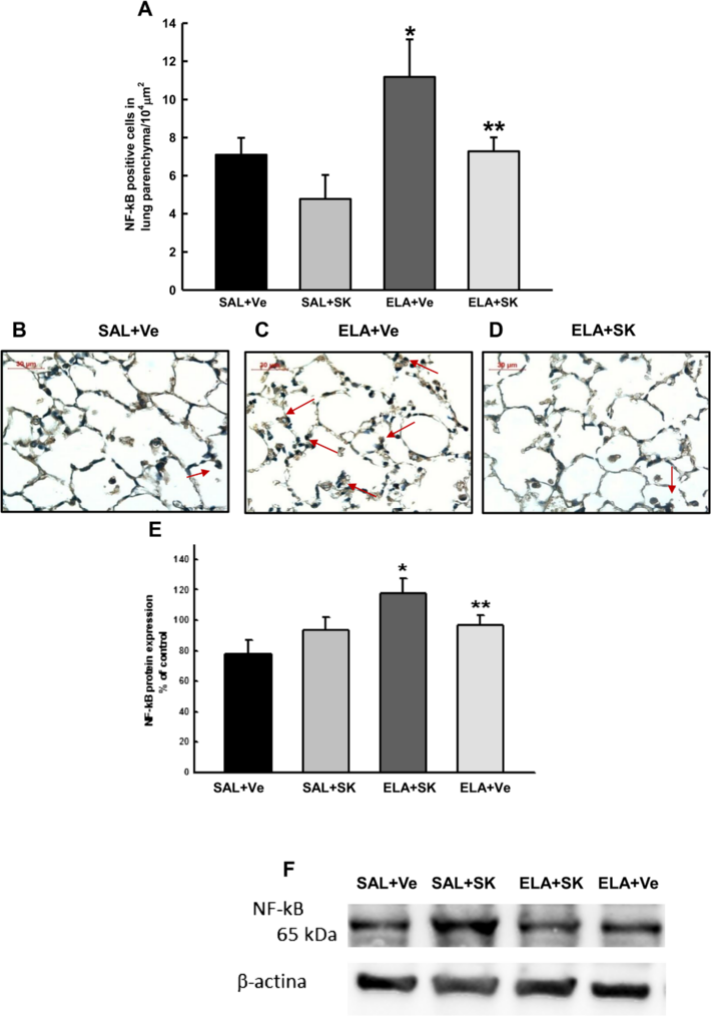
NF-κB in lung tissue. NF-κB-positive area (mean and SE) in the lung parenchyma **a**. Representative photomicrographs of the lung parenchyma immunostained for NF-κB **b-d**. Vehicle-treated animals that received elastase showed an increase in the NF-κB-positive area **c** compared with animals in the control group **b**. This response was attenuated in elastase animals by sakuranetin treatment **d**; compared with the ELA+Ve group **c**. NF-kB protein content and a representative Western blot of NF-kB **e-f**. The Western blot corroborates the immunohistochemistry data. *p < 0.05 compared with the SAL+Ve group; **p < 0.05 compared with the ELA+Ve group

We quantified the NF-κB-positive cells in lung tissue (Fig. 5a-d) by immunohistochemistry and determined the NF-κB protein content in lung homogenates by Western blotting (Fig. 5e-f). NF-κB expression, as examined by immunostaining and Western blotting, was higher in the animals that received elastase (ELA+Ve) compared with those that received saline (SAL+Ve) (*p* < 0.05). Treating the elastase-instilled animals with sakuranetin reduced both the number of NF-κB-positive cells and the NF-κB content (ELA+SK vs ELA+Ve; *p* < 0.05). Treatment with sakuranetin did not affect NF-κB expression in the control groups. Photomicrographs of the lung tissue immunostained for NF-κB are shown in Fig. 5b-d. Intense positive staining was observed in the animals that received elastase (c), and this positive staining was reduced by sakuranetin treatment (d). A representative blot is shown in Fig. 5f.

## Discussion

In the present study, we evaluated whether sakuranetin derived from the plant *Baccharis retusa* interferes with the inflammatory changes and destruction of lung tissue that is observed in an experimental murine emphysema model generated by the intranasal instillation of elastase. We showed that treatment with sakuranetin 2 h after elastase instillation attenuated lung inflammation, alveolar destruction and lung remodeling characterized by collagen and elastic fiber deposition. Furthermore, sakuranetin improved the imbalance between the expression of metalloproteinases (MMP-9 and MMP-12) and TIMP-1. One possible mechanism for sakuranetin activity could involve the regulation of NF-κB activation; sakuranetin decreased the expression of certain pro-inflammatory cytokines, including M-CSF, IL-1β and TNF-α, and regulated oxidative stress.

The animals that received elastase presented with emphysema, lung inflammation, increased collagen and elastic fiber deposition in the lung parenchyma and oxidative stress; these data confirm those from a previous study [18].

Sakuranetin was previously isolated and characterized [14–16] and shown to be the main component of the species *Baccharis retusa* (Asteraceae), a plant that is found in large quantities in southern Brazil. The dose of sakuranetin chosen in the present study was based on a previous report from our laboratory [16] and data from other *in vivo* models in which a dose–response curve was determined. In the current study, mice were treated with sakuranetin two hours after elastase instillation on day 0 and then again on days 7, 14 and 28. No adverse clinical effects were observed in the animals that received this treatment.

Corticosteroids are currently the principal treatment for COPD patients, particularly because both chronic bronchitis and emphysema are associated with lung inflammation. This class of drugs reduces at least some of the lung inflammation. However, little or no effect on lung destruction has been observed [17, 22]. Because lung destruction is the most important feature of emphysema, drugs that interfere with these alterations are relevant. In this study, we observed a reduction in alveolar destruction, as evidenced by the Lm index, in animals that received elastase and were treated with sakuranetin. This result suggests that this treatment may protect the lung against alveolar destruction, which is an important feature of emphysema. Although sakuranetin was administered 2 h after the elastase instillation, which does not simulate a treatment approach, Munoz-Barrutia et al. [23] used micro-CT chest imaging and pulmonary function tests to show enlargement of the airspaces within 1 h of elastase instillation.

Pulmonary inflammation is associated with the pathogenesis of COPD. The sakuranetin-treated animals had fewer total cells, macrophages, lymphocytes, and neutrophils in the BALF. Mainly, sakuranetin had an effect on the number of macrophages: 95 % of the cells in the BALF were macrophages. Consistent with our findings, other studies using elastase models have observed that approximately 95 % of the cells in the BALF were macrophages [18]. In addition, sakuranetin reduced the levels of M-CSF, which is correlated with the effects on macrophages [24]. Although neutrophils are central to the pathophysiology of COPD, the number of neutrophils in the BALF was quite low in the present study. Nevertheless, sakuranetin treatment reduced the number of neutrophils. Neutrophilic inflammation usually occurs after elastase instillation and is resolved after the first week [18, 25]. The time point evaluated in the present study (28 days) most likely explains the low number of neutrophils in the BALF.

Our data suggest that sakuranetin had an important anti-inflammatory effect in this model. However, although sakuranetin reduced lung inflammation by 50 %, the number of inflammatory cells was higher in the elastase- and sakuranetin-treated animals than in the non-emphysematous animals. Higher doses of sakuranetin or co-treatment with existing drugs, such as glucocorticoids, could produce more significant anti-inflammatory effects and should be the subject of future studies.

The anti-inflammatory effects of flavonoids have been shown in other models, and these effects most likely involve reducing the levels of pro-inflammatory cytokines and chemotactic factors and suppressing oxidative stress. According to Vlahos et al. [22], M-CSF is responsible for regulating the number of leukocytes during COPD pathogenesis. Kersul et al. [24] showed that the levels of IL-8, IL-6, IL-1β and TNF-α in sputum increase during emphysema exacerbation in humans. We showed that sakuranetin treatment reduced M-CSF, IL-1β and TNF-α levels, suggesting a regulatory effect on inflammatory cytokine levels. This effect could involve the ability of sakuranetin to regulate NF-κB activation, which in turn controls pro-inflammatory cytokine release, as suggested by Song et al. [26].

We previously showed that the effect of sakuranetin on allergic inflammation could also be due to effects on NF-κB regulation [16]. In the present study, sakuranetin potentially acted as an anti-inflammatory compound by inhibiting the expression of p65-NF-κB; sakuranetin treatment reduced both the number of NF-κB-positive cells in the lung parenchyma and NF-κB protein expression in the lung homogenates from the elastase-instilled animals. Importantly, the p65, c-REL, and RER-B subunits each possess a C-terminal transactivation domain; therefore, dimers of these aforementioned subunits are necessary to activate NF-κB gene transcription [27, 28], suggesting that sakuranetin attenuated the activation of p65-NF-κB.

Few studies have evaluated the effects of flavonoids in COPD. In this regard, Bras et al. [29] showed that polyphenolic compounds inhibit the activity of elastase. Culpitt et al. [30] showed that resveratrol, a component of red wine, reduces cytokine production by macrophages isolated from patients with COPD. Consistent with our results, Lixuan et al. [31] showed that baicalin reduces inflammation by inhibiting NF-κB in a cigarette smoke-induced model. There are no previous studies of sakuranetin in emphysema models.

Nevertheless, in terms of the effects on macrophages, sakuranetin did not reduce the levels of MCP-1 or MIP-2. These chemokines possess chemotactic properties that are important for the proliferation, differentiation and survival of lymphocytes, monocytes and basophils and participate in the activation and the late responses of neutrophils and monocytes [32].

Morphological changes in the collagen and elastic fibers are features of the pathophysiology of emphysema. Although lung remodeling associated with COPD has been studied for some years, it remains controversial, and the alterations in the lung structure of patients with emphysema have not been fully elucidated [33]. Structural changes in the ECM, including significant changes in the collagen, elastin and proteoglycan content, occur during the development of emphysema both in humans [33] and in experimental rodent models of this disease [18, 34]. It is known that the lung repair process may occur in a disorganized manner in individuals with emphysema [35, 33], and this feature contributes to the irreversible deterioration of lung function [34, 35].

Black et al. [5] demonstrated a reduction of approximately 18 % in elastic fibers in the lung parenchyma in emphysematous patients with various degrees of obstruction. Vlahovic et al. [36] studied lung biopsies from patients with emphysema and showed that the levels of collagen and elastic fibers were increased by approximately 3- or 4-fold compared with lungs from non-emphysematous patients. However, Spears et al. [37] observed a similar collagen content in lung strips from patients with COPD and in control subjects.

In our experimental model, there were increases in both collagen and elastic fibers 28 days after the elastase treatment. Similarly increased levels of elastic fibers in elastase-treated animals have been observed in other experimental models [18, 38]. It is possible, however, that this increase in elastic fibers is associated with the turnover of elastic fibers that are destroyed by the elastase instillation. We measured the total elastic fiber content without considering the structure of these fibers. Abnormal structure may be associated with the functional deterioration of these fibers, which can directly affect lung function. In this study, sakuranetin prevented the lung remodeling process in animals treated with elastase, as indicated by a reduction in the amount of collagen and elastic fibers in the alveolar septae.

The processes of ECM repair and degradation are known to be largely controlled by MMPs and their inhibitors. The expression of MMPs, particularly MMP-1, MMP-2, MMP-9 and MMP-12, is increased in patients with COPD [5], and this increase contributes to ECM degradation and the subsequent repair and turnover of fibers. Some authors have suggested that MMP-9 and MMP-12 are relevant to emphysema, are involved in experimental models of elastase [39], and are involved in the degradation of collagen fibers. The evaluation of MMP-9, MMP-12 and TIMP-1 expression in the inflammatory cells in the lung parenchyma revealed that sakuranetin reduced the number of MMP-9- and MMP-12-positive cells in emphysematous animals. MMP activity is regulated by TIMPs [40]. Therefore, we also assessed the expression of TIMP-1 in cells within lung tissue. Treatment with sakuranetin increased the number of TIMP-1-positive cells. This effect may be responsible for the reduction in MMP-9 and MMP-12 expression that was observed in the ELA+SK animals. Consistent with our findings, Ganesan et al. [41] showed that in mice with elastase/LPS-induced emphysema, quercetin treatment reduced MMP-9 and MMP-12 activity and increased the expression of SIRT-1, which regulates MMP expression. Together, these results suggest that sakuranetin regulates the remodeling process in this model primarily by modulating the balance between MMPs and TIMPs.

Increases in ROS levels and/or decreases in antioxidant levels induce oxidative stress. The response to oxidative stress is the major mechanism implicated in the development of pulmonary emphysema and is involved in the recruitment of inflammatory cells and the destruction of alveolar tissue [3]. To assess whether the effects of sakuranetin in this model were due to a reduction in oxidative stress, we evaluated the levels of 8-iso-PGF-2α [16], an acceptable marker of oxidative stress that is produced at the end of the oxidative reaction [42]. Sakuranetin reduced the 8-iso-PGF-2α-positive area, suggesting that the effects of sakuranetin in this experimental model can be at least partly attributed to its antioxidant activity. However, these data did not indicate whether the effects of sakuranetin involved reducing oxidants and/or increasing antioxidant substances. In agreement with our results, others have demonstrated that polyphenolic compounds can directly reduce oxidative stress *in vitro* and *in vivo* [11, 26].

Notably, we treated the animals with sakuranetin two hours after the elastase instillation. With respect to the mechanisms by which sakuranetin reduced the lung alterations in this model, it is possible that at this time, sakuranetin directly inhibited elastase activity, as has been described by others [29]; this could have induced slightly less lung injury and therefore reduced inflammation and remodeling. Although we have not evaluated the effects of sakuranetin on elastase activity, this should be considered a possibility. However, some authors have shown by micro-CT chest imaging that airspace enlargement has already occurred by one hour after elastase instillation [23], as discussed above. These data could suggest that the animals already had some degree of lung destruction when sakuranetin was administered.

In *in vivo* studies, it is difficult to exactly distinguish the direct or indirect effects of a tested compound. In experimental models of elastase-induced emphysema, elastase directly destroys the elastic fibers and the ECM, which in turn induces a repair process that involves recruiting inflammatory cells to the local injury site and regenerating the ECM components. Activated neutrophils release more elastase and generate reactive oxygen and nitrogen species that create a proteolytic *milieu*. Sakuranetin is a well-known antioxidant [43]; however, it also has anti-inflammatory effects because it inhibits NF-κB expression [16]. Therefore, we hypothesize that sakuranetin functions preventively by reducing oxidative stress and inhibiting NF-kB activation to reduce inflammation, which consequently attenuates the degradation of the alveolar septa. Furthermore, sakuranetin increased the expression of TIMP-1, which inhibits the activity of MMP-9, a metalloprotease that is involved in lung destruction. Although the exact effects should be further evaluated, the direct and/or indirect effects of sakuranetin on inflammation reduction have considerable clinical relevance.

Because the lung structure differs between humans and animal models, the data provided here should be interpreted carefully in terms of translating the effects to humans. Furthermore, is important to note that this emphysema model represents a mild form of the disease. In experimental models of cigarette smoke-induced emphysema, the inflammatory cell profile and the lung function alterations are different because chronic bronchitis is established first. In models of elastase-induced emphysema, the first alteration is alveolar tissue destruction. Finally, we did not detect any toxic effects of the flavonoid treatment; studies conducted with *Asteraceae* species did not show any hepatotoxic effects [44].

## Conclusion

In conclusion, we demonstrated that sakuranetin, a plant-derived flavonoid compound, attenuated lung inflammation, lung destruction and lung remodeling in a model of pulmonary emphysema. The inhibition of p65-NF-κB and pro-inflammatory cytokine expression, the reduction in oxidative stress responses and the improvement in the MMP/TIMP imbalance may account for these observations. Sakuranetin prevented the development of experimental emphysema; thus, it may represent a novel pharmacological tool that requires further investigation.

## Additional file

Additional file 1: **Supplementary material.**

## Abbreviations

BALF: bronchoalveolar lavage fluid
COPD: chronic obstructive pulmonary disease
ECM: extracellular matrix
Ers: respiratory system elastance
Gtis: tissue damping
Htis: tissue elastance
IL-1β: interleukin-1β
Lm: mean linear intercept
LREIMS: low resolution electronic impact mass spectrum
MCP: monocyte chemoattractant protein
M-CSF: macrophage colony-stimulating factor
MIP: macrophage inflammatory protein
MMP: matrix metalloproteases
NF-κB: nuclear transcription factor-kappa B
NMR: nuclear magnetic resonance
Raw: airway resistance
ROS: reactive oxygen species
Rrs: respiratory system resistance
TIMP: tissue inhibitors of metalloproteinases
TNF-α: tumor necrosis factor-α
