# Erratum: A flavanone from *Baccharis retusa* (Asteraceae) prevents elastase-induced emphysema in mice by regulating NF-κB, oxidative stress and metalloproteinases

**DOI:** 10.1186/s12931-015-0258-7

**Published:** 2015-09-17

**Authors:** Laura Taguchi, Nathalia M. Pinheiro, Clarice R. Olivo, Alessandra Choqueta-Toledo, Simone S. Grecco, Fernanda D. T. Q. S. Lopes, Luciana C. Caperuto, Mílton A. Martins, Iolanda F. L. C. Tiberio, Niels O. Câmara, João Henrique G. Lago, Carla M. Prado

**Affiliations:** Department of Biological Science, Universidade Federal de São Paulo, Rua Artur Riedel, 275 - Eldorado, Diadema, SP Brazil; Department of Exact and Earth Sciences, Universidade Federal de São Paulo, Diadema, Brazil; Department of Medicine, Faculdade de Medicina da Universidade de São Paulo, São Paulo, Brazil; Department of Immunology, Biological Institute, Universidade de São Paulo, São Paulo, Brazil

## Erratum

After publication of the original article [1] it came to the author’s attention that FAPESP were not acknowledged as the agency who financed the study. The authors wish to thank the contribution of FAPESP.
