# Supplementary material for: A flavanone from Baccharis retusa (Asteraceae) prevents elastase-induced emphysema in mice by regulating NF-κB, oxidative stress and metalloproteinases
Source: Respir Res. 2015 Jun 30;16(1):79. doi: 10.1186/s12931-015-0233-3 (PMC4489216; doi:10.1186/s12931-015-0233-3)
Supplement: Supplementary file 1 — Supplementary material. [file 12931_2015_233_MOESM1_ESM.docx]

**SUPPLEMENTARY MATERIAL**

**A flavanone from Baccharis retusa (Asteraceae) prevents elastase-induced emphysema in mice by regulating NF-κB, oxidative stress and metalloproteinases**

1. **Nuclear Magnetic Resonance (NMR) and low-resolution electronic impact mass spectrometry (LREIMS) data of sakuranetin**


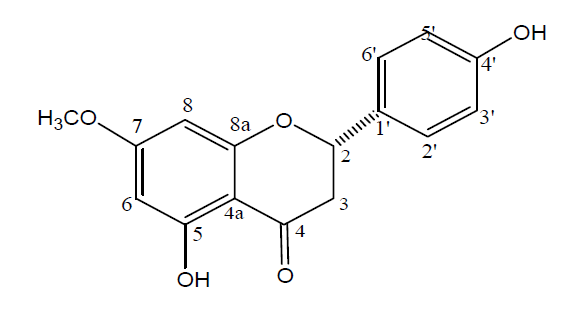


**Figure 1.** Chemical structure of 5,4’-dihydroxy-7-methoxy-flavanone (sakuranetin)

LREIMS (70 eV) *m/z* (int. rel.): 286 (67), 193 (33), 180 (39), 167 (100), 138 (24), 120 (44), 95 (38), 69 (25). ^1^H NMR (300 MHz, CDCl_3_ + CD_3_OD) δ_H_: 7.26 (d, *J* = 8.5 Hz, H-2’/H-6’), 6.83 (d, *J* = 8.5 Hz, H-3’/H-5’), 6.01 (s, H-6/H-8), 5.32 (dd, *J* = 13.0 and 3.0 Hz, H-2), 3.77 (s, OCH_3_-7), 3.08 (dd, *J* = 17.2 and 13.0 Hz, H-3a), 2.73 (dd, *J* = 17.2 and 3.0 Hz, H-3b). ^13^C NMR (75 MHz, CDCl_3_ + CD_3_OD) δ_C_: 196.5 (C-4), 168.0 (C-4’), 163.6 (C-7), 163.0 (C-5), 157.4 (C-9), 129.0 (C-1’), 127.7 (C-2’/C-6’), 127.6 (C-6), 115.3 (C-3’/C-5’), 102.8 (C-10), 93.9 (C-8), 79.1 (C-2), 55.3 (OCH_3_), 42.8 (C-3).


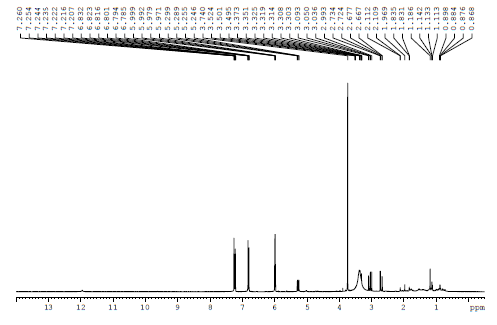


**Figure 2**. ^1^H NMR spectrum of sakuranetin (δ, CDCl_3_ + CD_3_OD, 300 MHz)


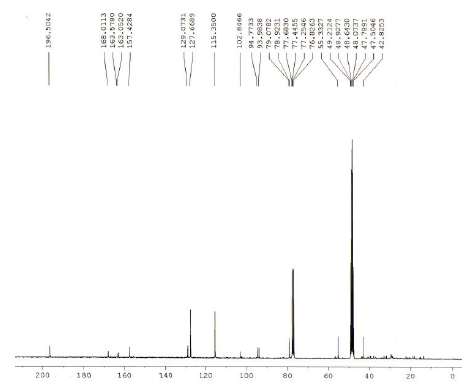


**Figure 3**. ^13^C NMR spectrum of sakuranetin (δ, CDCl_3_ + CD_3_OD, 75 MHz)


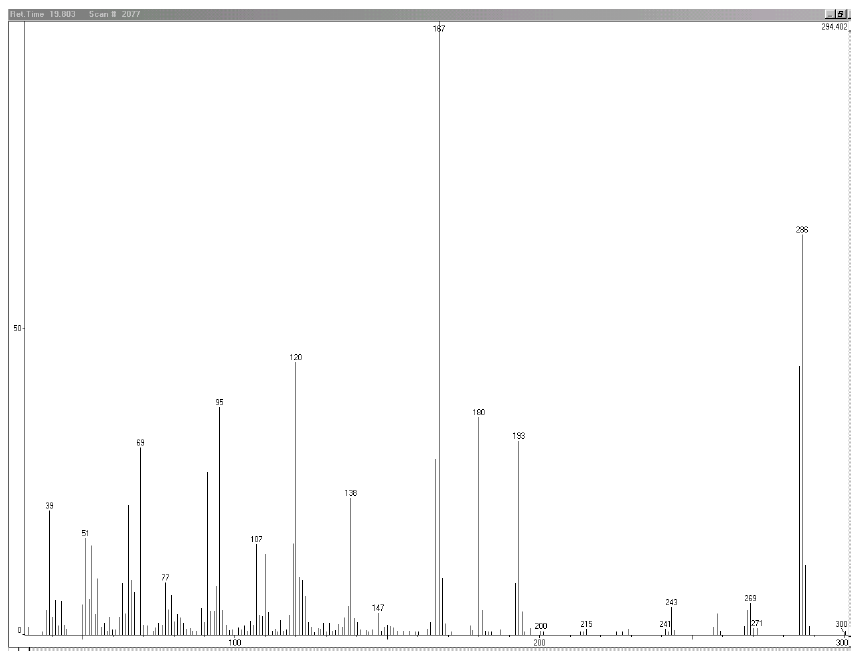


**Figure 4.** LREIMS (70 eV) spectrum of sakuranetin

1. **Vehicle Treatment (DMSO plus saline) did not interfere in any lung responses**

Because DMSO is an organo-sulfur compound that possesses both toxic effects and antioxidant effects, we first evaluated whether DMSO treatment (vehicle) interfered with the lung inflammation (5A) and with 8-iso-PGF-2α (5B) expression by comparing animals treated with vehicle (DMSO+Saline) with animals that did not receive any treatment. There were no significant differences in the lung inflammation [SAL: 1.05±0.17; SAL+Ve: 0.73±0.06; ELA: 3.71±1.06; ELA+Ve: 3.94±0.31] nor in the 8-iso-PGF-2α positive area [SAL: 14.77±1.00; SAL+Ve: 10.53±1.83; ELA: 31.01±3.40; ELA+Ve: 33.69±2.55] (evaluated by t-test) between animals treated or not with vehicle (Fig 5). The differences observed in ANOVA were between ELA and ELA+Ve compared with SAL and SAL+Ve (P<0.05) showing the effects of elastase increasing total cells and oxidative stress. Therefore, we used the animals treated with vehicle as controls for the sakuranetin treatment.


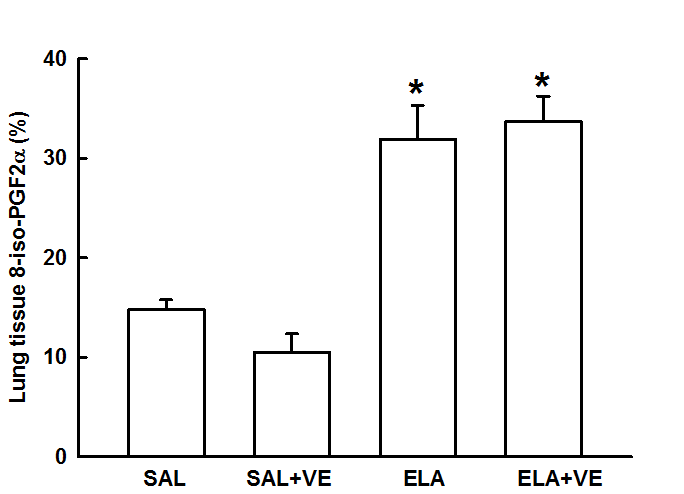

**Figure 5.** Total Cells counted in BALF (A) and lung tissue 8-iso-PGF2a measured by immunohistochemistry in lung evaluated in animals that received saline or elastase and were not treated or treated with vehicle (DMSO+Saline). No differences were observed between animals treated with vehicle or animal that was not treated (T-test between SAL and SAL+VE or ELA and ELA+VE). *P<0.05 compared to SAL and SAL+VE groups, respectively.
